# Synergy between an emerging monopartite begomovirus and a DNA-B component

**DOI:** 10.1038/s41598-021-03957-7

**Published:** 2022-01-13

**Authors:** Alassane Ouattara, Fidèle Tiendrébéogo, Nathalie Becker, Cica Urbino, Gaël Thébaud, Murielle Hoareau, Agathe Allibert, Frédéric Chiroleu, Marie-Stéphanie Vernerey, Edgar Valentin Traoré, Nicolas Barro, Oumar Traoré, Pierre Lefeuvre, Jean-Michel Lett

**Affiliations:** 1 Laboratoire de Virologie et de Biotechnologies Végétales, Institut de l’Environnement et de Recherches Agricoles (INERA), 01 BP 476, Ouagadougou 01, Burkina Faso; 2 CIRAD, UMR PVBMT, 97410 St Pierre, La Réunion France; 3 Université de La Réunion, UMR PVBMT, 97410 Saint-Pierre, La Réunion France; 4 Université Joseph Ki-Zerbo, 03 BP 7021, Ouagadougou 03, Burkina Faso; 5 Laboratoire Mixte International Patho-Bios, IRD-INERA, 01 BP 476, Ouagadougou 01, Burkina Faso; 6 UMR Institut de Systématique, Évolution, Biodiversité (ISYEB), Muséum National d’Histoire Naturelle, CNRS, Sorbonne Université, EPHE, Université des Antilles, 57 rue Cuvier, CP 50, 75005 Paris, France; 7 CIRAD, UMR PHIM, 34090 Montpellier, France; 8 PHIM Plant Health Institute, INRAE, Univ Montpellier, CIRAD, Institut Agro, IRD, Montpellier, France; 9 Laboratoire National de Biosécurité (LNB), 06 BP 10798, Ouagadougou 06, Burkina Faso

**Keywords:** Ecology, Evolution, Microbiology

## Abstract

In recent decades, a legion of monopartite begomoviruses transmitted by the whitefly *Bemisia tabaci* has emerged as serious threats to vegetable crops in Africa. Recent studies in Burkina Faso (West Africa) reported the predominance of pepper yellow vein Mali virus (PepYVMLV) and its frequent association with a previously unknown DNA-B component. To understand the role of this DNA-B component in the emergence of PepYVMLV, we assessed biological traits related to virulence, virus accumulation, location in the tissue and transmission. We demonstrate that the DNA-B component is not required for systemic movement and symptom development of PepYVMLV (non-strict association), but that its association produces more severe symptoms including growth arrest and plant death. The increased virulence is associated with a higher viral DNA accumulation in plant tissues, an increase in the number of contaminated nuclei of the phloem parenchyma and in the transmission rate by *B. tabaci*. Our results suggest that the association of a DNA-B component with the otherwise monopartite PepYVMLV is a key factor of its emergence.

## Introduction

Multipartite virus genomes, packaged in separate particles and accounting for a large proportion of all plant viruses, have shown undeniable evolutionary success, despite the possible costs and benefits of such genomic organization (for a review, see Sicard et al.^1^). One of the advantages of a multi-component genome is an increased ability to exchange information modules, thereby enabling new genetic combinations possibly with new biological features favourable to their emergence and dissemination^1,2^.

Geminiviruses are plant viruses with circular, single-stranded (ss) DNA genomes encapsidated in twinned icosahedral particles^3,4^. They are divided into 14 genera based on host range, insect vector, genome organization and a classification based on pairwise nucleotide identities coupled with phylogenetic support. Whereas 13 of the genera (*Becurtovirus, Capulavirus, Citlodavirus, Curtovirus, Eragrovirus, Grablovirus, Maldovirus, Mastrevirus, Mulcrilevirus, Opunvirus, Topilevirus, Topocuvirus* and *Turncurtovirus*) consist of viruses with monopartite genomes only^4,5^, the currently most extensively described genus *Begomovirus* harbours viruses with either one or two genomic components (respectively referred to as monopartite or bipartite). Begomoviruses are also frequently associated with one or more smaller satellites^6,7^, providing an additional layer of genomic plasticity. The component known as DNA-A is homologous to the genomes of all geminiviruses and encodes proteins required for movement (MP/C4 for monopartite begomoviruses), replication, control of gene expression, overcoming of host defences, encapsidation and insect transmission^3^. Bipartite begomoviruses harbour a second component, whose ancestral origin remains unclear^8^, it is referred to as DNA-B, encodes two proteins (BC1 and BV1) with functions in intra- and intercellular movement in host plants, and contributes to the increase in viral load and disease severity^8,9^. Importantly, within DNA-A and DNA-B components, a homologous intergenic region (IR), referred to as the common region (CR), contains the origin of replication: a conserved hairpin structure, with an upstream Rep binding iteron sequence^3,10^. The similarity of the CRs ensures binding between the DNA-A-encoded Rep and the cognate DNA-B. Furthermore, *in natura* CR recombinations have been described between DNA-A & -B components^11^.

Since 1980s, begomoviruses have emerged in many areas of the world and extensively described in tomato (*Solanum lycopersicum*)^12^, they have become a major constraint in the production of vegetables. In Africa, a complex of at least 20 monopartite begomovirus species are involved in tomato yellow leaf curl or tomato leaf curl diseases (TYLCD and ToLCD), including seven species described in West Africa^13,14^. Among them, the species *Pepper yellow vein Mali virus* has been identified as the most prevalent and severe tomato-infecting begomovirus in tomato and pepper in Burkina Faso^15^. Interestingly, whereas pepper yellow vein Mali virus (PepYVMLV) was originally described as a Western African monopartite begomovirus^16,17^, it has frequently been found associated with a DNA-B component^15^. The vast majority of the components of tomato-infecting bipartite begomoviruses have an obligate relationship^18,19^. However, this obligate relationship appears to be absent for both tomato yellow leaf curl Thailand virus (TYLCTHV) and tomato leaf curl Gujarat virus (ToLCGV) DNA-A components because they are able to induce systemic and symptomatic infections in the host plants, *Nicotiana benthamiana*^20^ and in tomato^21^, respectively, in the absence of their cognate DNA-B. These data suggest that these viruses represent evolutionary intermediates between monopartite and bipartite begomoviruses.

To understand the contribution of the DNA-B component to the biology of the otherwise monopartite PepYVMLV, we evaluated biological traits related to their virulence, virus accumulation and their location in plant cellular tissue, as well as transmission of the virus by mechanical-, *agrobacterium*- and whitefly-mediated inoculation. We demonstrated that even though the DNA-B component is not essential for infection, it increases viral accumulation and the number of infected nuclei, the virulence and the transmission rate of PepYVMLV by *Bemisia tabaci*. Taken together, our results suggest that the recruitment of a DNA-B component by the monopartite PepYVMLV is a key epidemiological factor that has enabled PepYVMLV to become the most prevalent virus responsible for the most severe viral disease of tomato crops in Burkina Faso.

## Results

### Higher infectivity and acute virulence of PepYVMLV DNA-A associated with DNA-B

**Figure 1:**
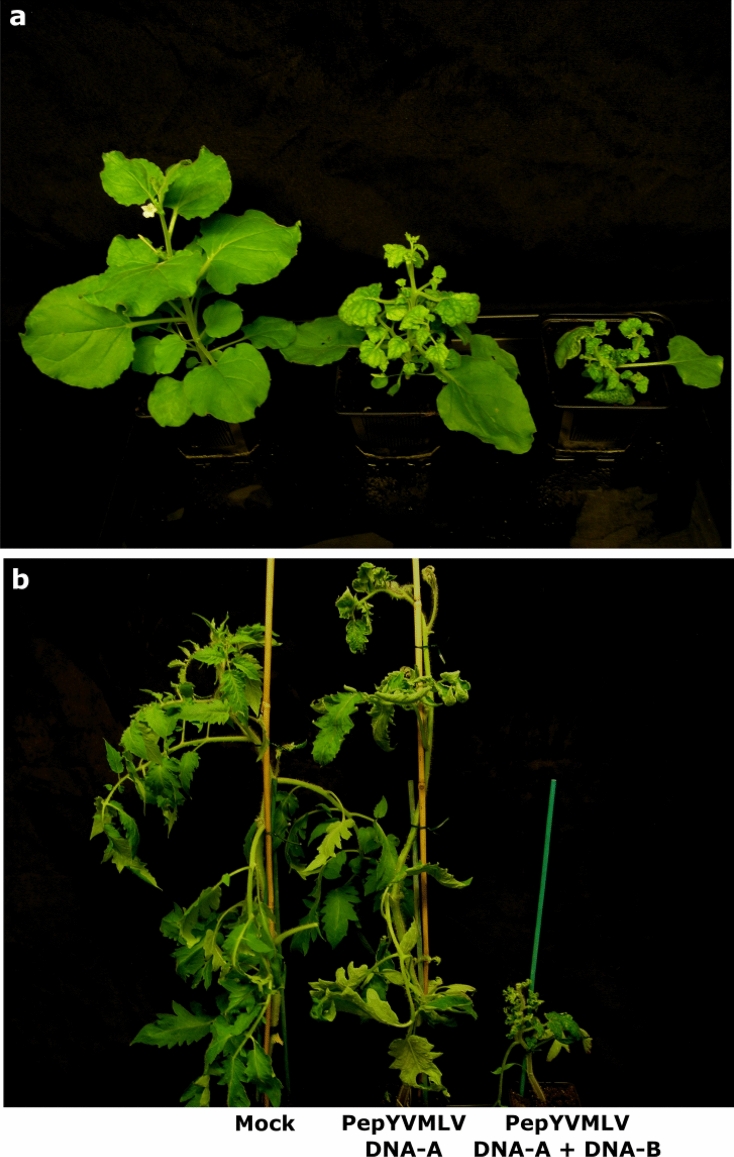
Disease symptoms in (**a**) *Nicotiana benthamiana* and (**b**) tomato (*Solanum lycopersicum*) plants agroinoculated with mock, PepYVMLV in single (DNA-A) or mixed (DNA-A and -B) infection at 29 days post inoculation. Distinct symptoms of leaf crumpling with yellowing and stunting were observed between single (mild symptoms) and mixed (very severe symptoms) infections on both *N. benthamiana* and tomato plants.

*N. benthamiana* and *S. lycopersicum* (tomato) plants agro-inoculated with PepYVMLV DNA-A alone or associated with DNA-B developed strikingly distinct symptoms of leaf crumpling with yellowing and stunting (Fig. 1a,b). *N. benthamiana* plants agro-inoculated with PepYVMLV DNA-A (of which 92.5% tested positive for DNA-A using conventional PCR) developed mild symptoms in 82.5% of inoculated plants (Table 1, Fig. 1a). In contrast, in mixed agroinoculation with DNA-B, very severe symptoms were observed in *N. benthamiana* (of which 100% tested PCR-positive for DNA-A & B) with plant growth arrest in 85% of inoculated plants. When tomato plants were agro-inoculated with PepYVMLV DNA-A, 20% developed very mild symptoms (Table 1, Fig. 1b), even though viral DNA-A was detected by PCR in 80% of inoculated plants. In contrast, in mixed agroinoculation with DNA-B, very severe symptoms were observed in tomato plants (of which 87% tested PCR-positive for DNA-A & B), with growth arrest in 95% and death in 17% of inoculated plants (Table 1, Fig. 1b). As positive control, tomato plants inoculated with the highly infectious TYLCV-IL DNA-A in single or mixed infection with DNA-B (of which 100% tested PCR-positive for TYLCV-IL) developed typical symptoms of TYLCV disease (Table 1). Interestingly, DNA-B was detected by PCR in 28% of mixed agroinoculated tomato plants with TYLCV-IL. Control plants (PepYVMLV DNA-B or mock-agroinoculated) remained asymptomatic.

**Table 1:** Infectivity of PepYVMLV and TYLCV-IL DNA-As in single or mixed infection with PepYVMLV DNA-B after agroinoculation of *N. benthamiana* and tomato plants.

	Single infection		Mixed infection		
	Symptom	PCR DNA-A	Symptom^a^	PCR DNA-A	PCR DNA-B
*N. benthamiana/*PepYVMLV	82.5% (33/40)	92.5% (37/40)	85% (34/40)	100% (40/40)	100% (40/40)
Tomato/PepYVMLV	20% (12/60)	80% (48/60)	95% (57/60) [10]	100% (60/60)	87% (52/60)
Tomato/TYLCV-IL	100% (60/60)	100% (60/60)	100% (60/60)	100% (60/60)	28% (17/60)

^a^Square brackets: number of dead plants at the end of the experiment (32 dpi).

### Higher symptom progression and severity for PepYVMLV DNA-A associated with DNA-B

**Figure 2:**
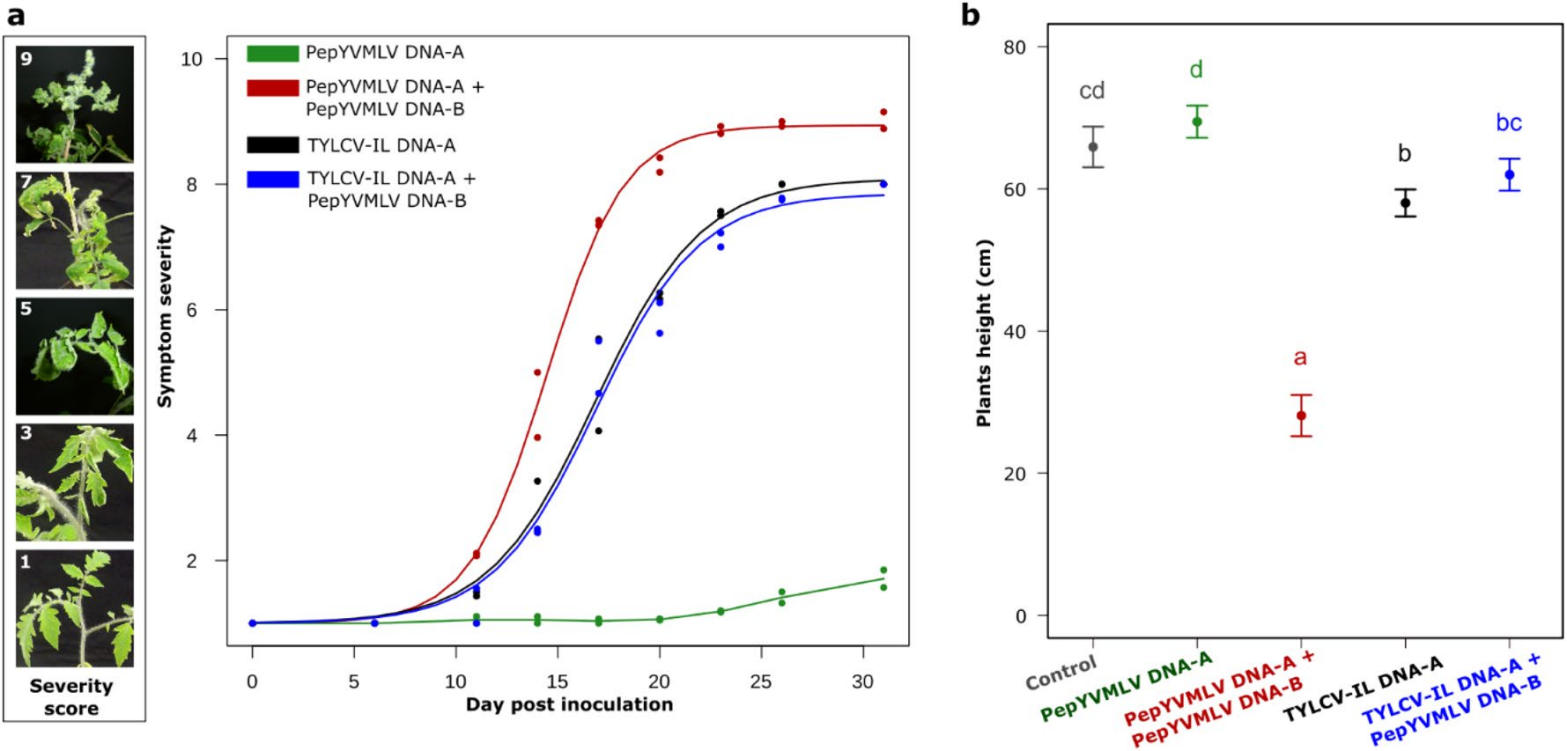
(**a**) Kinetics of estimated symptom severity of tomato (yellow) leaf curl disease following agroinoculation of tomato plants with PepYVMLV DNA-A and TYLCV-IL DNA-A in single (green and black lines) or mixed (red and blue lines) infection with DNA-B, respectively. Each point represents the average for one experiment (n = 30). The symptom severity scale (left scale bar) ranges from 1 (no symptoms) to 10 (plant death). (**b**) Mean height of tomato plants after agroinfection with PepYVMLV DNA-A or TYLCV-IL in single or mixed infection with DNA-B at 32 days post inoculation. For each dot, vertical bars represent 95% confidence intervals. Identical letters on top of the bars indicate groups with non-significant differences in height.

The kinetics of symptom severity of PepYVMLV DNA-A and TYLCV-IL DNA-A were compared in single or mixed infection with DNA-B (Fig. 2a). Tomato plants agroinoculated with PepYVMLV DNA-A & B exhibited symptoms of leaf crumpling, yellowing and stunting at 12 days post inoculation (dpi) (Fig. 2a). In contrast, tomato plants agroinoculated only with PepYVMLV DNA-A exhibited their first symptoms, which were similar but far less severe, at 27 dpi. Typical symptoms of leaf curling, yellowing and dwarfism were observed in tomato plants agroinoculated with TYLCV-IL DNA-A in single or mixed infection with DNA-B from 12 dpi, although less severe than those induced by PepYVMLV DNA-A & B. Symptom severity increased exponentially, and then linearly, before reaching a plateau with very severe disease symptoms at 22 dpi for PepYVMLV DNA-A co-inoculated with DNA-B, and 10 days later for TYLCV-IL in single or mixed agroinfection. The progression of symptom severity (parameter A; i.e. the slope of the linear phase at the inflection point), the time to reach 50% of the maximum severity (parameter B) and the severity at the plateau phase (parameter C) differed significantly between PepYVMLV DNA-A & B and TYLCV-IL DNA-A infection (*p* = 0.0054, *p* < 10^–4^ and *p* = 2 × 10^–4^, respectively; Table 2). No significant difference was observed in the virulence kinetics of TYLCV-IL associated or not with DNA-B (*p* = 0.6, Table 2).

**Table 2:** Estimated parameters [95% confidence intervals] of the logistic growth model of the progression of disease symptom severity in tomato plants agroinoculated with PepYVMLV and TYLCV-IL DNA-A in single or mixed inoculation with PepYVMLV DNA-B.

Virus^a^	Parameter estimates for the logit model		
	**A**	**B**	**1 + C**
PepYVMLV DNA-A + DNA-B	0.53 [0.46–0.60]	14.46 [14.17–14.74]	8.94 [8.74–9.13]
TYLCV-IL DNA-A	0.38 [0.30–0.46]	16.85 [16.20–17.50]	8.09 [7.69–8.49]
TYLCV-IL DNA-A + DNA-B	0.39 [0.30–0.49]	16.89 [16.17–17.62]	7.85 [7.41–8.29]

A: Slope of the linear phase at the inflection point of the logistic disease progression curve.

B: Time to reach 50% of the symptom severity at the plateau phase.

1 + C: Maximum symptom severity at the final plateau.

^a^For PepYVMLV DNA-A alone, symptom severity does not show a logistic progress curve (see Fig. 2a); thus, parameters A, B and C cannot be calculated in this case.

### Negative effect of the DNA-B component on tomato growth

Thirty-two days post-agroinoculation, no significant difference in size was observed between the controls and plants inoculated with PepYVMLV DNA-A alone (*p* = 0.428, Fig. 2b). Conversely, a significant difference in size (*p* = 0.001) was observed between the control and plants agroinoculated with TYLCV-IL alone. The agroinoculation of PepYVMLV DNA-A and -B to tomato plants strongly affected their growth, with a notable reduction in size compared to plants inoculated with PepYVMLV DNA-A alone (*p* < 10^–4^, Fig. 2b). In contrast, no significant difference in size was observed between plants agroinoculated with TYLCV-IL DNA-A, in single or mixed inoculation with PepYVMLV DNA-B (*p* = 0.264).

### Higher accumulation of PepYVMLV DNA-A in association with DNA-B

**Figure 3:**
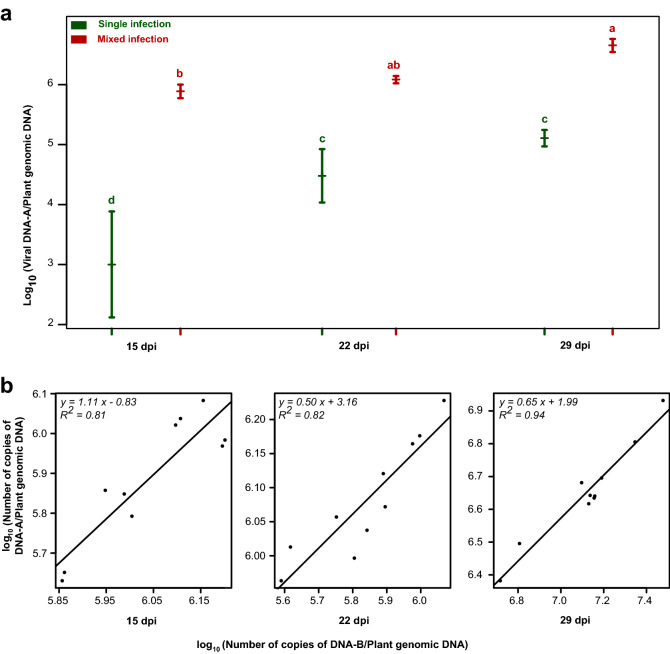
SYBR Green real-time PCR quantifications. (**a**) Average accumulation of PepYVMLV in single (DNA-A) or mixed (DNA-A and -B) infection of tomato plants (*Solanum lycopersicum*) at 15, 22 and 29 days post inoculation (dpi). (**b**) The bottom panels represent the linear correlation between PepYVMLV DNA-A and -B normalized loads. Vertical bars represent 95% confidence intervals. Identical letters on top of the bars indicate groups with non-significant differences in height.

Within-plant accumulation of PepYVMLV DNA-A, associated or not with the DNA-B component, was evaluated in agroinoculated tomato plants using SYBR Green real-time PCR assay (Fig. 3a). Experiments were conducted on separate sets of ten plants. At 15, 22 and 29 dpi, the DNA-A molecules accumulated more in mixed infections than in single infections (264, 25 and 35-fold, respectively; *p* < 0.001). For all real-time PCR assays, positive linear relationships were found between the normalized copy numbers of PepYVMLV DNA-A and DNA-B (Fig. 3b). Similar results were obtained at 32 dpi in an independent experiment (data not shown), using TaqMan real-time PCR assays.

### Higher transmission rate of PepYVMLV DNA-A in association with DNA-B

Two transmission experiments were performed independently using synchronous adult female individuals of the cryptic species Middle East-Asia Minor 1 (MEAM1) of *B. tabaci,* fed on tomato plants agroinoculated with PepYVMLV DNA-A alone or in association with DNA-B (Table 3). Based on PCR PepYVMLV DNA-A detection, transmission rates of respectively, 80% and 83% for PepYVMLV DNA-A alone, reached significantly higher values (100%) for PepYVMLV DNA-A and DNA-B in both mixed infection experiments (*p* = 4 × 10^–5^). The evaluation of transmission rates based on disease symptoms confirmed the difference between single and mixed infections (*p* < 10^–4^), with transmission rates of respectively, 52% and 40% for PepYVMLV DNA-A alone, and respectively, 71% and 69% for PepYVMLV DNA-A and DNA-B together.

**Table 3:** Transmission rates of PepYVMLV in single or mixed infection with DNA-B by synchronous females of *Bemisia tabaci* MEAM1.

	Single infection		Mixed infection		
	Symptoms	PCR DNA-A	Symptoms	PCR DNA-A	PCR DNA-B
Experiment 1	52% (24/46)	80% (37/46)	71% (29/41)	100% (41/41)	98% (40/41)
Experiment 2	40% (14/35)	83% (29/35)	69% (27/39)	100% (39/39)	100% (39/39)

### No sap transmission of PepYVMLV DNA-A associated with DNA-B on tomato

The sap transmission capacity of PepYVMLV DNA-A, associated or not with the DNA-B component, was evaluated on *N. benthamiana* and tomato plants. Inoculum was collected from agroinoculated tomato plants with typical disease symptoms in which the presence of DNA-A and/or DNA-B had been confirmed by PCR. We observed a single infection with PepYVMLV DNA-A, in association with DNA-B (1/30), in *N. benthamiana* (Supp. table 2). We were unable to detect any transmission of PepYVMLV by plant sap to tomato plants, regardless of the genomic components used.

### Higher number of contaminated phloem parenchyma cells in mixed infection

**Figure 4:**
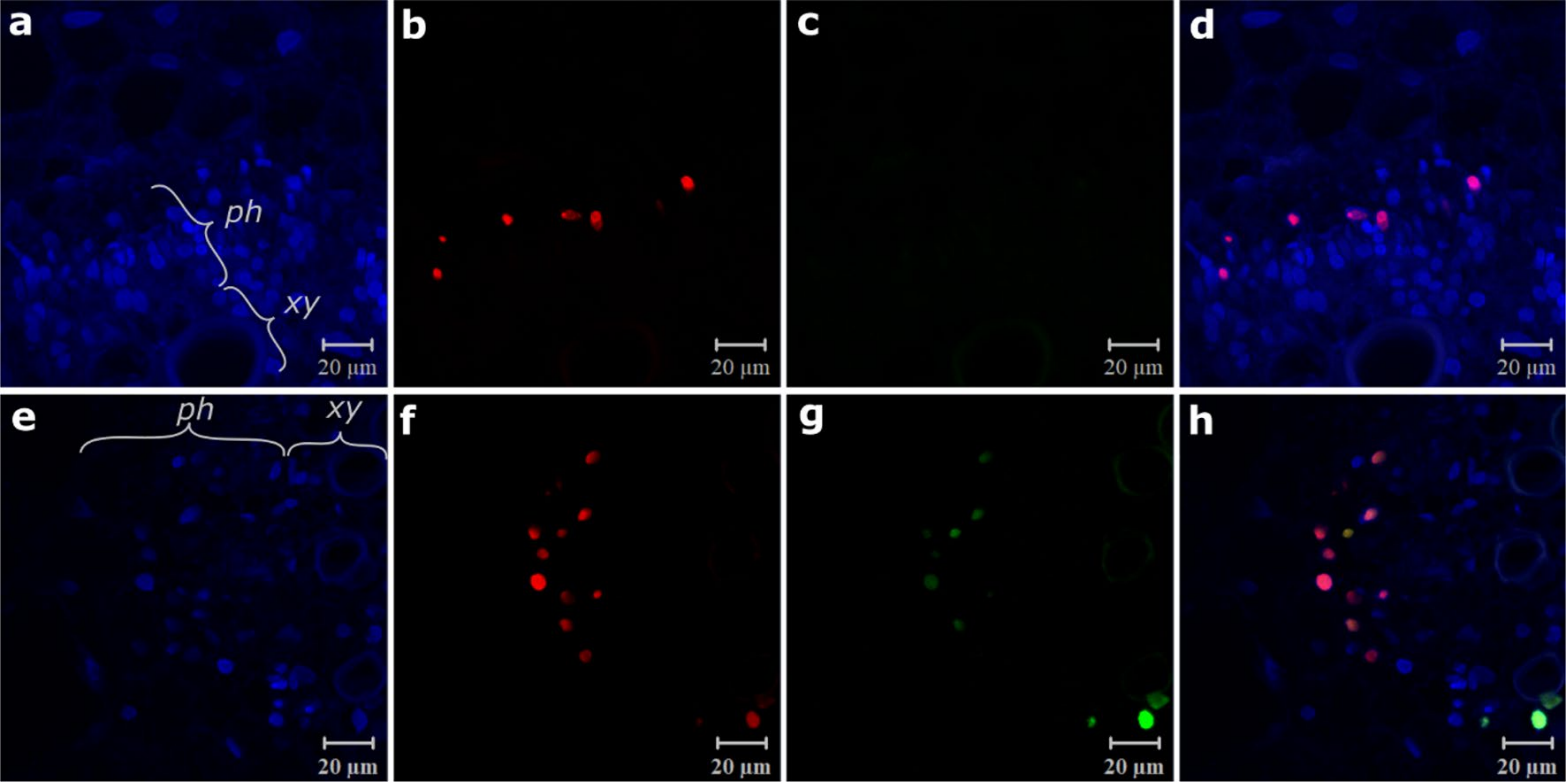
Location of PepYVMLV DNA-A in single (**a**–**d**) or mixed (**e**–**h**) infection with DNA-B in cross sections of infected tomato petioles. DNA-A (**b**,**f**) and DNA-B (**c**,**g**) components revealed by red and green FISH probes, respectively. Nuclei are stained with DAPI blue. Images (**d**,**h**) in which the three channels (blue, red, and green) are merged enable identification of the components located together in the cell nuclei. Phloem (ph) and xylem (xy) bundles are indicated.

First, immunofluorescence observations (356 cells out of a total of 49 cross sections), using the nuclear stain DAPI and PepYVMLV-DNA-A & B probes, showed that both DNA-A and DNA-B were exclusively located in the nuclei of phloem parenchyma cells in both single and mixed infections (Fig. 4a–h). Second, analysis of the images corresponding to mixed infections showed that the two components DNA-A and DNA-B were mostly located together (50–78% of DNA-A and/or -B infected cells, Table 4, Fig. 4e–h). Third, consistent with the greater accumulation of PepYVMLV DNA-A in the presence of DNA-B previously observed in real-time PCR assays, we observed a significantly higher number of contaminated cells in mixed infection (DNA-A and/or -B, *p* < 0.001), regardless of the sampling date (Table 4). The number of cells labelled only with DNA-A were similar at 15 and 22 dpi (*p* > 0.1) but were significantly fewer in mixed infection at 29 dpi (*p* < 0.001). A minority of cells with only DNA-B were observed in mixed infections (8–14% of infected cells, Fig. 4c,g, and Table 4).

**Table 4:** Number of infected cells per cross section of tomato petioles infected by PepYVMLV DNA-A in single or mixed infection with DNA-B.

dpi	Infection	NCSPA	Mean number of infected cells per cross section of petioles					
			DNA-A		DNA-B	DNA-A and -B	DNA-A and/or -B	
15	Single	10	1 ± 1	*p* = 0.9	0	0	1 ± 1	*p* < 10^–3^
	Mixed	8	2 ± 2 (22%)		0 (0%)	7 ± 3 (78%)	9 ± 2	
22	Single	8	4 ± 1	*p* = 0.16	0	0	4 ± 1	*p* < 10^–3^
	Mixed	7	5 ± 1 (36%)		2 ± 2 (14%)	7 ± 2 (50%)	14 ± 2	
29	Single	10	7 ± 1	*p* < 10^–3^	0	0	7 ± 1	*p* < 10^–3^
	Mixed	6	2 ± 2 (17%)		1 ± 1 (8%)	9 ± 2 (75%)	13 ± 3	

DNA-A and -B components were detected respectively using red- and green-FISH probes.

Values in brackets show the percentage of infected cells per detected component (DNA-A or -B) or components (DNA-A and -B).

*dpi* days post inoculation, *NCSPA* number of cross sections of petioles analysed.

*p* values were obtained from multiple comparisons of means of cell numbers between single and mixed infection cases using post-ANOVA Tukey HSD-test.

## Discussion

Recent studies in Burkina Faso reported the identification of at least five begomovirus species in tomato crops^16,17,22,23^, with the predominance of PepYVMLV. Interestingly, this species of viruses was frequently associated with a previously uncharacterized DNA-B component^15^. To assess whether the DNA-B component is associated with the emergence of PepYVMLV as the currently most prevalent and most severe plant virus disease of tomato crops in Burkina Faso, we evaluated biological traits related to virulence, virus accumulation in the plant and transmission.

*Agrobacterium*-mediated inoculation experiments showed that PepYVMLV DNA-A alone induced systemic and symptomatic infections in *N. benthamiana* and tomato plants (Fig. 1, Table 1). This result is consistent with PepYVMLV DNA-A genomic organization as a typical Old World monopartite begomovirus^24,25^, and with the role of the V1 ORF in cell-to-cell movement^26^. Such a non-strict association between a monopartite begomovirus and a DNA-B component has been previously described for ToLCGV^27^ and TYLCTHV^20^ which, in the absence of their cognate DNA-B, are able to induce systemic and symptomatic infections both in the experimental host *N. benthamiana*^20^ and in tomato^27^. In contrast, the New World bipartite begomoviruses absolutely require their cognate DNA-B for systemic movement and for the development of symptoms^5^. Taken together, these experimental results suggest that non-strict bipartite begomoviruses may represent evolutionary intermediates between monopartite and bipartite begomoviruses.

Unlike single agroinoculation of tomato plants with PepYVMLV, mixed agroinoculation with PepYVMLV DNA-A and -B not only strongly increased symptoms of leaf crumpling, yellowing and stunting, as observed in the field, but also led to the death of 17% of inoculated plants under controlled conditions in a climatic chamber. Several studies reported that begomovirus DNA-B contributes to symptom production^27,28^ and that BC1 protein is a determinant of pathogenicity^29,30^. This extreme virulence might be a “maladaptive” consequence of the recent association between PepYVMLV DNA-A and DNA-B. If the observed increase in transmission is offset in the long term by a reduced transmission from infected hosts with a shorter lifespan, virulence may decrease in the future^31^. If not, and if coinfections by PepYVMLV DNA-A and DNA-B are frequent, the countries affected this new association would be facing a sort of ‘Darwinian Demon’ with both high virulence and high transmissibility.

TYLCV-IL has been reported to be one of the most severe and devastating tomato viruses worldwide^32^. In this study, we compared PepYVMLV and TYLCV-IL virulence in association or not with DNA-B. Although when inoculated with TYLCV-IL, some plants also reached maximum severity (score = 9) at 32 dpi, the overall severity score was higher for PepYVMLV associated with DNA-B (earlier appearance of disease symptoms, higher mean symptom score at plateau, higher impact on plant growth). Taken together, these observations demonstrate that PepYVMLV associated with DNA-B is more virulent in tomato in controlled conditions than our TYLCV-IL isolate, and underscores the new global risk of PepYVMLV for the tomato crop if it were to spread beyond West Africa.

The quantification of within-plant accumulation of PepYVMLV DNA-A in single or mixed infection with DNA-B in tomato plants showed that mixed-infected plants contained more PepYVMLV DNA-A than in single infection, and that the copy numbers of the two genomic components presented a positive linear relationship. At the cellular level, FISH analyses of infected plants suggest that this higher viral accumulation is accompanied by a significantly higher proportion of infected phloem cells (DNA-A and/or -B). BV1 and BC1 proteins, encoded by DNA-B which have an analogous function in viral movement to that of the V1/C4 proteins of the monopartite begomoviruses^26^, have been reported to facilitate the escape of some bipartite begomoviruses from the phloem, as well as to infect non-phloem tissues^3^. This has been observed in particular for a strict bipartite geminivirus from the New World, bean dwarf mosaic virus, which is not phloem-limited and was detected in most cell types of the inoculated host leaves^33^. However, in our experiments, PepYVMLV DNA-A, in single or mixed infection with DNA-B, is detected exclusively in the phloem parenchyma of tomato.

In contrast to its significant impact on PepYVMLV infection, DNA-B had no such impact either on TYLCV-IL infection rate or on symptom severity. Interestingly, DNA-B was detected by PCR in only 28% of mixed agroinfected tomato plants, indicating a non-optimal association between TYLCV-IL DNA-A and DNA-B (Table 1). Geminiviruses replicate by a rolling circle mechanism initiated by the binding of the virus-DNA-A encoded replication-associated protein (Rep)^8,10,34^ to the Rep binding iteron sequence. DNA-A and DNA-B components of bipartite begomoviruses display similar iteron sequences, thereby ensuring that the DNA-A-encoded Rep can initiate replication of both components. Mutation analyses revealed the importance of the conservation of the first three bases of the iteron sequences in the efficient initiation of replication by Rep. Iteron sequences TYLCV-IL (GGTGTCT) and PepYVMLV DNA-B (GGGGTAC) may thus be poorly compatible^35^.

Transmission is a crucial step in the life cycle of pathogens as it ensures their spread and maintenance in host populations. The immobile nature of plants, together with the pectin and cellulose barrier around the cells, have constrained most plant virus to depend on vectors (mainly insects) to exit, transfer, and enter another host^36^. Contact transmission from one plant to the other has only rarely been reported for begomoviruses, and mechanical transmission with infected sap has been demonstrated for some begomovirus species in experimental conditions^37^. Interestingly, in the case of the tomato leaf curl New Delhi virus (ToLCNDV), a bipartite begomovirus, the DNA-B component was already reported to be implicated in mechanical transmission^37^. Our experimental mechanical transmission (negative for all conditions except a single *Nicotiana* plant) excluded the potential ability of PepYVMLV DNA-B for significant contact transmission. On the other hand, the data collected in our study indicate that the presence of the DNA-B component increases insect transmission of the virus. Indeed, we demonstrated that PepYVMLV DNA-A accumulates more within tomato plants and is significantly better transmitted by whitefly in mixed infection with DNA-B than in single infection (1.2 times more, with transmission reaching 100% in the presence of DNA-B). Despite the scarcity of studies, a positive relation between within-plant viral load and vector transmission rate is widely accepted for plant viruses^32,38^.

## Conclusion

Our study highlights the role of a DNA-B component in the virulence and transmission of a monopartite begomovirus. The high prevalence and severity of PepYVMLV in tomato crops in Burkina Faso is probably due to the fitness advantage gained through the recruitment of a DNA-B component by the monopartite PepYVMLV. In the case of the non-strict association of PepYVMLV DNA-A and DNA-B, the latter may be regarded as an “extra-genomic viral component” that can bolster the pathogenicity, accumulation, and transmission of its cognate virus, while itself depending on the associated virus for successful infection. At the agroecosystem level, when associated with DNA-B, PepYVMLV DNA-A has been recovered from a wide range of hosts^15^, including some weeds that are frequently found in fields. Maintenance of the virus in alternative hosts present in the cultivated area between epidemics enables it to survive the seasonal cycle of tomato cultivation, and may contribute to the predominance of PepYVMLV associated with DNA-B.

## Materials and methods

### Construction of infectious clones

Full-length DNA-A and DNA-B molecules of PepYVMLV [Burkina Faso:Sakabi:Pepper72:2013] ([BF:Sak:Pe72:13], EMBL: MH778694/MK092768) previously cloned into pGEM-3Zf (Promega, USA)^15,24^, were used for the construction of infectious clones in the binary vector pCambia0380 (Cambia, Australia). A 456-bp *Pst*I/*Bam*HI-digested fragment comprising the IR of the DNA-A was cloned to generate a 0.16-mer (pCambia0380-0.16). The full-length monomer was then cloned into *Bam*HI-digested pCambia0380-0.16 to generate a 1.16-mer of PepYVMLV DNA-A. For the DNA-B, a 2057-bp *Eco*RI/*Bam*HI-digested fragment comprising the IR was cloned to generate a 0.77-mer (pCambia0380-0.77). The full-length monomer was cloned into *Bam*HI-digested pCambia0380-0.77 to generate a 1.77-mer of PepYVMLV DNA-B. Recombinant plasmids were transferred from *Escherichia coli* strain JM-109 cells into *Agrobacterium tumefaciens* (strain C58) by triparental mating using *E. coli* HMB101 harbouring the helper plasmid pRK2013^39^. Along with PepYVMLV DNA-A and DNA-B, an agroinfectious clone of the Israel strain of tomato yellow leaf curl virus (TYLCV-IL)^32^ was used as a virulent control.

### Agrobacterium-mediated inoculation experiments

*Agrobacterium tumefaciens* harbouring PepYVMLV DNA-A, PepYVMLV DNA-B or TYLCV-IL DNA-A were grown in liquid culture medium for 48 h and adjusted to an OD_600nm_ of 1.3 before inoculation. Tomato plants (*Farmer 209*, Known-You Seed) and *Nicotiana benthamiana* were mono-inoculated (PepYVMLV DNA-A, PepYVMLV DNA-B or TYLCV-IL DNA-A) or bi-inoculated (PepYVMLV DNA-A + PepYVMLV DNA-B or TYLCV-IL DNA-A + PepYVMLV DNA-B) at the three-leaf stage by injecting about 50 µL of *A. tumefaciens* culture. For mixed inoculations, *Agrobacterium* cultures were mixed in equal volumes. A total of 60 tomato and 40 N*. benthamiana* plants were agroinoculated per condition. In addition, 20 plants each of tomato and *N. benthamiana* were punctured with sterile needles and used as negative controls. Inoculated plants were then arranged in a complete random block design and maintained for 32 days in an insect-proof growth chamber at 25 ± 4 °C with a 12 h photoperiod and 70 ± 10% relative humidity. Symptoms were scored twice a week until 30 days post inoculation (dpi). The symptom severity scale ranged from 1 (no symptom) to 10 (plant death), with grades 1–9 corresponding to the scale of Lapidot et al.^40^. Plant size was measured at 32 dpi to assess the effect of viral infection on plant growth. Apical leaves were collected for the detection and the quantification of viral genomes using PCR and real-time PCR, respectively, as described below. Two replicates were performed at different dates for the experiments carried out to assess virulence.

### Mechanical inoculation experiments

Symptomatic tomato leaves were collected from agroinoculated tomato plants, frozen at − 80 °C and ground into a fine powder using a pestle and a mortar with liquid nitrogen, as previously described^41^. Tomato and *N. benthamiana* seedlings were inoculated by rubbing the leaves with the resulting sap mixed with carborundum powder. All inoculated plants were maintained in the growth conditions described above. Negative controls were mock-inoculated plants. Symptoms were assessed 30 days later, and the plants were tested for the presence of viral DNA by PCR.

### Whitefly-mediated inoculation experiments

A non-viruliferous *B. tabaci* colony of the cryptic species MEAM1 (formerly biotype B) was reared on cabbage plants (*Brassica oleracea*), in a growth chamber at 25 °C in the day and 20 °C at night, with 70% relative humidity and a 12-h photoperiod. Viruliferous whiteflies were obtained after a 72-h acquisition access period (AAP) on tomato plants agroinoculated in single or mixed infections with PepYVMLV DNA-A and PepYVMLV DNA-B. After the AAP, adult females were collected based on morphological criteria, mainly the size of the abdomen, verified under binocular and a single insect was than deposited on each healthy tomato seedlings (*Farmer 209*, Known-You Seed) at the one-leaf growth stage, and then placed under micro-cages for a 72-h inoculation access period (IAP). At the end of the IAP, insects were manually removed, and the tomato seedlings were sprayed with insecticide (Confidor®, Bayer). In order to discard insects with an unknown IAP, only plants on which the insect had been found alive were used for the rest of the experiment (PepYVMLV DNA-A, n = 80 plants; PepYVMLV DNA-A + DNA-B, n = 81 plants). Negative controls were mock-inoculated plants (non-viruliferous whiteflies, n = 20 plants). The plants were maintained in the same growth conditions as those described above. After 30 days, symptoms were assessed, and plants were tested for the presence of viral DNA by PCR.

### Virus detection

Total DNA was extracted from 20 mg of plant material, previously dehydrated in an oven at 50 °C for 48 h, using the DNeasy Plant Miniprep Kit (Qiagen) according to the manufacturer’s instructions, with two successive 50 µL elutions with ultrapure water. Extracts were stored at − 20 °C before use. Conventional PCR was carried out to detect viral DNA in samples collected at 32 dpi and 30 dpi in agroinoculation and whitefly-mediated inoculation experiments, with specific TYLCV-IL^42^ and PepYVMLV DNA-A and -B primer sets^15^.

### Fluorescence in situ hybridization (FISH)

#### Preparation of the probes

Segments of 85–90 nucleotides of PepYVMLV DNA-A and DNA-B were amplified by PCR using the GoTaq Polymerase kit (Promega) with specific primers PepYVMLV-A-F/PepYVMLV-A-R and PepYVMLV-B-F/PepYVMLV-B-R according to Ouattara et al*.* (2020)^15^. PCR products were then migrated in a 1% agarose gel and purified using NucleoSpin® Gel and PCR Clean-up (Macherey Nagel). The resulting amplicons were then used as templates to produce segment-specific probes using the Invitrogen and Alexa Fluor Bioprime DNA Labelling Kit (Alexa Fluor 488 and 568) as described elsewhere^41^.

#### Sample preparation and in situ hybridization

Petiole samples from agroinoculated plants were collected at 15, 22 and 29 dpi. Immediately after sampling, the samples were fixed in a phosphate-buffered saline 1 × solution with 4% paraformaldehyde (PFA) and 0.2% Tween-20. Fixed samples were embedded in 8% low melting agarose in a 24-well plate before sectioning with a vibratome (MICROM). Hybridization was performed as previously described^41^.

#### Microscopy observations

A total of 49 cross sections of tomato petioles were observed by microscopy, comprising 21 and 28 cross-sections from tomatoes infected with PepYVMLV DNA-A in single and mixed infection with DNA-B, respectively. All observations were made using an LSM700 Confocal Microscope (ZEISS) with ZEN software following the protocol of Vernerey et al.^41^. In practice, parameters were adjusted to obtain sufficient resolution and fluorescence intensity signal recovery in a chosen series of infected plant exhibiting a high intensity of fluorescence without saturation points. Images were taken with the 40 × water immersion objective at a resolution of at least 512 × 512 with a pinhole aperture of 1 Airy Unit so as to work in confocal mode. Three sequential tracks were set, one for each fluorochrome used (using lasers at 405 nm for DAPI, 488 nm for Alexa Fluor 488, and 555 nm for Alexa Fluor 568). Analyses were performed using maximum intensity projections so that all the fluorescence emitted by all the nuclei was accounted for. These microscopic images were analysed using Image J software.

### Within-plant virus quantification

Agroinoculated plants sampled at 15, 22 and 29 dpi were used. Each plant sample consisted of five 4-mm-diameter leaf disks collected from the youngest leaves and stored at − 80 °C until analysis. DNA was extracted as described elsewhere^43^. The proportions of PepYVMLV DNA-A and -B were quantified using SYBR Green Real-Time PCR as described by Urbino et al.^43^. Primers were designed and used to quantify the viral molecules in infected plants (for sequences and conditions of use, see Supplementary table 1). Real-time PCR was performed in a 10-µL reaction mix comprising the 2 × LightCycler® 480 SYBR Green I Master kit (Roche, Germany), each primer, and two microliters of a 1/100 dilution of the DNA template. Plant genomic DNA of each extract was quantified using the nuclear-encoded large subunit ribosomal RNA gene (*S. lycopersicum* L. 25S ribosomal RNA gene) as described by Conflon et al.^44^. The amplification reactions were run in 384-well optical plates in Roche LightCycler System (Roche, Germany). Amplification conditions were 95 °C for 10 min followed by 40 cycles of 10 s at 95 °C, 30 s at 60 °C and 20 s at 72 °C. Two quantification replicates were performed per sample.

### Statistical analysis

All statistical analyses were performed using the R statistical software^45^. Nonlinear regression analyses between the copy number of the DNA-A and DNA-B components were performed, testing different link functions (Cauchy, cloglog, logistic, logit, loglog and probit), to fit the progression of disease severity with *gnls* function, using the package nmle^46^. Based on the likelihood and using Akaike’s information criterion (AIC), the logit function was selected as the best model. In this model, written y ~ 1 + C/(1 + exp(− A * (x − B))), the disease severity (y) is dependent on the dpi (x) and three biologically relevant parameters where A is the slope of the linear phase at the inflection point, 1 + C is the disease severity at the plateau phase, and B is the time to reach 50% of disease severity at the plateau phase. The estimated parameters were then compared between the different conditions using likelihood ratio tests in nested models. For these analyses, only plants were used for which single (DNA-A) or mixed (DNA-A and -B) infections were validated by PCR. Real-time PCR data were expressed as the log of the ratio of the quantity of virus DNA to that of plant DNA. The amount of the DNA-A component in single and mixed inoculation conditions was compared using an ANOVA *F*-test. For mixed infections, linear regression was used to estimate the correlation between the copy numbers of DNA-A and DNA-B component copy numbers. FISH data were subjected to multiple comparisons of means using post-ANOVA Tukey HSD-test^47^.

### Ethical statement

All the experimental protocols involving plants adhered to relevant ethical parameters/ regulations.

## Supplementary Information

Supplementary Information 1.

Supplementary Information 2.
