# Supplementary material for: Synergy between an emerging monopartite begomovirus and a DNA-B component
Source: Sci Rep. 2022 Jan 13;12:695. doi: 10.1038/s41598-021-03957-7 (PMC8758689; doi:10.1038/s41598-021-03957-7)
Supplement: Supplementary file 1 — Supplementary Information 1. [file 41598_2021_3957_MOESM1_ESM.docx]

**Supplementary table 1:** Primers developed and used in SYBR Green Real-Time PCR.

| **Name** | **5′-3′ sequence** | **nM** |
| --- | --- | --- |
| PepYVMLV-A_1259F | GTCCAGACCTTGAAGTTGAGA | 500 |
| PepYVMLV-A_1344R | ATCCTCACACTCCAAATACG | 500 |
| PepYVMLV-B_521F | ATGTCCTAAACTACCAGTACG | 500 |
| PepYVMLV-B_610R | CCTGTCTGGATCTCCTCAATA | 500 |
