# Supplementary material for: Synergy between an emerging monopartite begomovirus and a DNA-B component
Source: Sci Rep. 2022 Jan 13;12:695. doi: 10.1038/s41598-021-03957-7 (PMC8758689; doi:10.1038/s41598-021-03957-7)
Supplement: Supplementary file 2 — Supplementary Information 2. [file 41598_2021_3957_MOESM2_ESM.docx]

**Supplementary table 2:** Evaluation of sap transmission capacity of PepYVMLV DNA-A and/or DNA-B on *Nicotiana benthamiana* and tomato plants 40 days after inoculation.

|  | ***Nicotiana benthamiana*** | | | ***Solanum lycopersicum* (tomato)** | | |
| --- | --- | --- | --- | --- | --- | --- |
| **Diagnosis** | **Symptoms*** | **PCR^¤^** | | **Symptoms*** | **PCR^¤^** | |
|  |  | **DNA-A** | **DNA-B** |  | **DNA-A** | **DNA-B** |
| **DNA-A** | 0 (0/30) | 0 (0/10) | 0 (0/10) | 0 (0/30) | 0 (0/10) | 0 (0/10) |
| **DNA-A + DNA-B** | 3 (1/30) | 5 (1/20) | 5 (1/20) | 0 (0/30) | 0 (0/10) | 0 (0/10) |
| **DNA-B** | 0 (0/30) | 0 (0/10) | 0 (0/10) | 0 (0/30) | 0 (0/10) | 0 (0/10) |
| **Control** | 0 (0/30) | 0 (0/10) | 0 (0/10) | 0 (0/30) | 0 (0/10) | 0 (0/10) |

*Percentage (rounded off to the unit) of begomovirus-like symptoms (symptomatic samples/total)

^¤^Percentage (rounded off to the unit) of PepYVMLV DNA-A or DNA-B (PCR-positive samples/total)
